# Real-world experience and prognostic factors in ovarian carcinosarcoma: a single-center retrospective study from China

**DOI:** 10.3389/fonc.2025.1577139

**Published:** 2025-06-10

**Authors:** Wanwan Ji, Yunmei Zhuo, Xianzhong Cheng

**Affiliations:** ^1^ Department of Surgery, Jiangsu Cancer Hospital, Jiangsu Institute of Cancer Research, The Affiliated Cancer Hospital of Nanjing Medical University, Nanjing, China; ^2^ Department of Chemotherapy, Jiangsu Cancer Hospital, Jiangsu Institute of Cancer Research, The Affiliated Cancer Hospital of Nanjing Medical University, Nanjing, China; ^3^ Department of Gynecologic Oncology, Jiangsu Cancer Hospital, Jiangsu Institute of Cancer Research, The Affiliated Cancer Hospital of Nanjing Medical University, Nanjing, China

**Keywords:** ovarian carcinosarcomas, prognostic factors, PARP inhibitors, homologous recombination deficiency, breast cancer gene

## Abstract

**Objective:**

Ovarian carcinosarcomas (OCS) is a rare type of ovarian cancer. Due to its low incidence, studies are limited to several case reports/case series and small-scale retrospective study. We carried out this study to explore prognostic factors and treatment strategies for OCS.

**Methods:**

Patients diagnosed with OCS between March 2012 and October 2023 at Jiangsu Cancer Center were enrolled in this study. Baseline Characteristics, treatment strategies and survival of all enrolled patients were recorded. Kaplan-Meier analysis with a log-rank Mantel-Cox test was used to compare progression-free survival (PFS) between different groups.

**Results:**

Twenty-six patients met inclusion criteria. The median PFS of all enrolled patients was 17.53 months. We firstly demonstrated that patients with ascites ≥500 ml (27.83 months *vs.* 13.7 months, *p*=0.12, HR 0.72), age ≥58 years (22.93 months *vs.* 13.53 months, *p*=0.354, HR 0.62), diameter of tumor<10cm (27.83 months vs. 12.80 months, *p*=0.095, HR 0.36), Ki-67 ≥70% (22.93 months *vs.* 13.53 months, *p*=0.093, HR 0.39) had a trend of better prognosis. Five patients underwent genetic testing, 4 of whom were homologous recombination deficiency (HRD)-positive and treated with PARP inhibitor (PARPi). The median PFS of the 4 patients was 22.68 months.

**Conclusions:**

Our study demonstrated that age at diagnosis, diameter of tumor, Ki-67 index, and volume of ascites may be prognostic factors of OCS. Patients with HRD positive/BReast CAncer gene (BRCA) mutation may benefit from PARPi.

## 1 Introduction

Ovarian cancer is the sixth leading cause of cancer-related death in women (1). Ovarian carcinosarcomas (OCS), also known as malignant mixed Mullerian tumors (MMMT), aggressive type of epithelial ovarian neoplasm, represent 1%-3% of all histologic subtypes of ovarian malignancies and remain poorly understood (2, 3). Hyeong In Ha et al. revealed that the age-standardized incidence rates (ASRs) of ovarian carcinosarcoma was 0.064 per 100,000 women in Korea between 1999 and 2018 (4). Besides, Barnholtz-Sloan et al. reported that 13,643 women were diagnosed with primary invasive ovarian cancer, and 382 (2.8%) of the women had ovarian carcinosarcoma between 1988 and 1997, using data from the SEER Program (5). Histologically, OCS consists of both high-grade carcinomatous and sarcomatous elements (6, 7). It is an uncommon form of gynecological cancer associated with high morbidity and mortality, and the prognosis continues to be dismal (8, 9).

A review of the Surveillance, Epidemiology, and End Results (SEER) Program data from 1998 to 2009 reported that patients with OCS have consistently poorer prognosis than those with high grade serous carcinoma of the ovary (10). Previous case series and small-scale study have also noted that poor prognosis in women with OCS has been associated with overriding sarcomatoid element more than 25% (11), overexpression of vascular endothelial growth factor (VEGF) (12), p53 mutation (13, 14), Ki-67 overexpression (15), older age (16, 17), advanced stage (16, 18), and bulk residual disease after surgery (19, 20). Nevertheless, there is no consensus about these factors.

Due to low incidence of this disease, there are few clinical studies on ovarian carcinosarcoma, with only some case reports and case series reported. Therefore, we conducted this single-center retrospective study to provide some reference for the diagnosis and treatment of OCS.

## 2 Materials & methods

### 2.1 Study design and population

We retrospectively reviewed clinicopathological data from patients diagnosed and treated at Jiangsu Cancer Center between March 2012 and October 2023. The study enrolled patients with histologically confirmed ovarian carcinosarcomas, including ovarian carcinosarcomas, fallopian tube carcinosarcomas and primary peritoneal carcinosarcoma. Inclusion criteria were: (1) age ≥ 18 years; (2) confirmed diagnosis of ovarian carcinosarcomas, fallopian tube carcinosarcomas and primary peritoneal carcinosarcoma. We excluded patients who did not receive treatment at our center. This study was approved by the Ethics Committee of the Affiliated Cancer Hospital of Nanjing Medical University. All enrolled patients signed the informed consent.

### 2.2 Data collection

Patient data including age at diagnosis, primary location, histopathological type, the International Federation of Gynecology and Obstetrics (FIGO) stage, tumor size, the timing of surgery (primary or interval debulking surgery), surgical outcome, BRCA/HRD status and subsequent treatment strategies. All data were collected from medical records and follow-up information. All patients were followed up through outpatient visits or by phone. Progression-free survival (PFS) data were obtained based on imaging examinations. The last follow-up occurred in June 2024. Disease progression was evaluated by computed tomography (CT)/magnetic resonance imaging (MRI) or positron emission tomography imaging (PET/CT). Clinical response was defined in accordance with the standards of the Response Evaluation Criteria in Solid Tumors (RECIST1.1) (21). The pathology of all patients was initially reviewed by at least two pathologists from Jiangsu Institute of Cancer Research.

### 2.3 Outcomes

Date of diagnosis was defined as date of first visit to hospital. PFS was defined as time from diagnosis to first evidence any of the following: appearance of new disease via radiographic imaging or clinical exam, elevation in CA125 above the normal range, or patient death from any cause. Short-term efficacy was evaluated by RECIST 1.1. Kaplan-Meier analysis with a log-rank Mantel-Cox test was used to compare progression-free survival (PFS) between different groups. In addition, univariate COX analysis and multivariate COX regression analysis were also attempted in this study, but due to the low incidence rate and the small number of included subjects, these two methods were not adopted. Statistical analysis was performed using IBM SPSS Statistics v.27.0 software and Graphpad Pism9.5 (version 9.5).

## 3 Results

**Figure 1:**
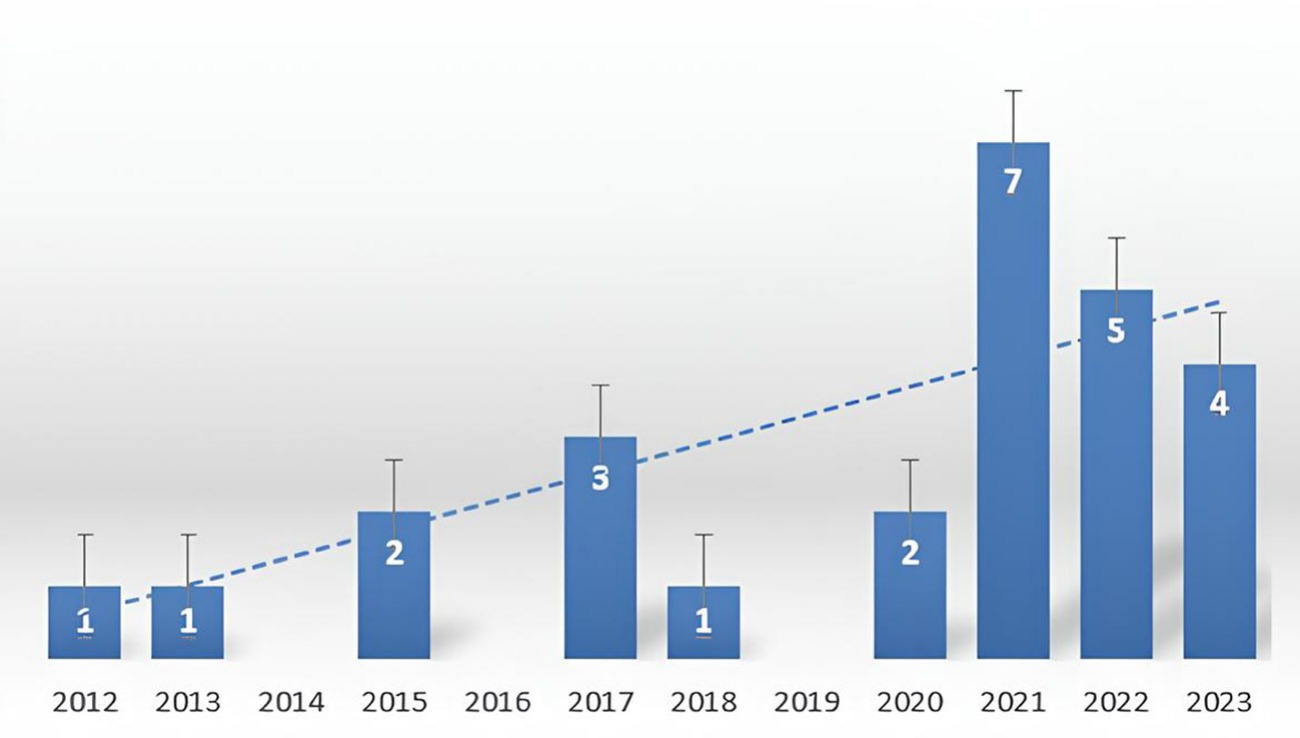
The patients diagnosed with OCS in each year at our cancer center between March 2012 and October 2023.

A total of 26 patients met the inclusion criteria in this study. The patients diagnosed in each year was shown in **Figure 1**. The detailed clinical characteristics of the included patients are presented in **Table 1**. All included patients were of Han ethnicity, and the mean age at diagnosis was 58 years (range 45 and 79 years). There were 25 patients (96.2%) diagnosed with ovarian cancer and 1 patient (3.8%) diagnosed with fallopian tube cancer. In terms of FIGO stage at diagnosis, 3 patients were stage I at diagnosis (11.5%), 3 patients were stage II (11.5%), 15 patients were stage III (57.7%), and 5 patients were stage IV (19.2%). A total of 5 patients underwent genetic testing, of which 2 patients had BRCA mutations and 4 patients were HRD positive. All patients underwent cytoreductive surgery, including 19 patients (73.1%) with primary debulking surgery (PDS) and 7 patients (26.9%) with interval debulking surgery (IDS). As respect to surgical outcome, 22 (84.6%) achieved optimal cytoreductive surgery, 3 (11.5%) achieved suboptimal outcome and 1 (3.8%) was not recorded. Twenty-five patients received chemotherapy after surgery (96.2%), and only 1 patient received Chinese medicine treatment (3.8%). The clinicopathological characteristics of all enrolled patients were presented in **Table 1**.

**Table 1:** Baseline clinicopathological characteristics of included patients.

Characteristics	Number of patients (percent)
Age, yrs	
Median age (range)	58 (45-79)
≤58	13 (50.0)
>58	13 (50.0)
Primary tumor location	
Ovary	25 (96.2)
Fallopian tube	1 (3.8)
Carcinomatous elements	
Serous carcinoma	10 (38.5)
Endometrioid carcinoma	5 (19.2)
Mucinous adenocarcinoma	3 (11.5)
Clear cell carcinoma	1 (3.8)
Uncategorized carcinom	7 (26.9)
FIGO stage	
I	3 (11.5)
II	3 (11.5)
III	15 (57.7)
IV	5 (19.2)
Unilateral/bilateral	
Unilateral	16 (61.5)
Bilateral	10 (38.5)
Maximum diameter	
≤10cm	16 (61.5)
>10cm	6 (23.1)
unknown	4 (15.4)
Ascites	
Yes	14 (53.8)
No	12 (46.2)
Family history of cancer	
Yes	7 (26.9)
No	19 (73.1)
ECOG	
0	11 (42.3)
1	15 (57.7)
Type of surgery	
PDS	18 (69.2)
IDS	8 (30.8)
Outcome of primary debulking surgery	
R0	17 (65.4)
R1	5 (19.2)
R2	3 (11.5)
Unknown	1 (3.8)
PARPi treatment	
Yes	4 (15.4)
No	22 (84.6)

yrs, years; FIGO, International Federation of Gynecology and Obstetrics; ECOG, Eastern Cooperative Oncology Group; PDS, Primary debulking surgery; IDS, Interval debulking surgery; PARPi Poly (ADP-ribose), polymerase inhibitor.

**Figure 2:**
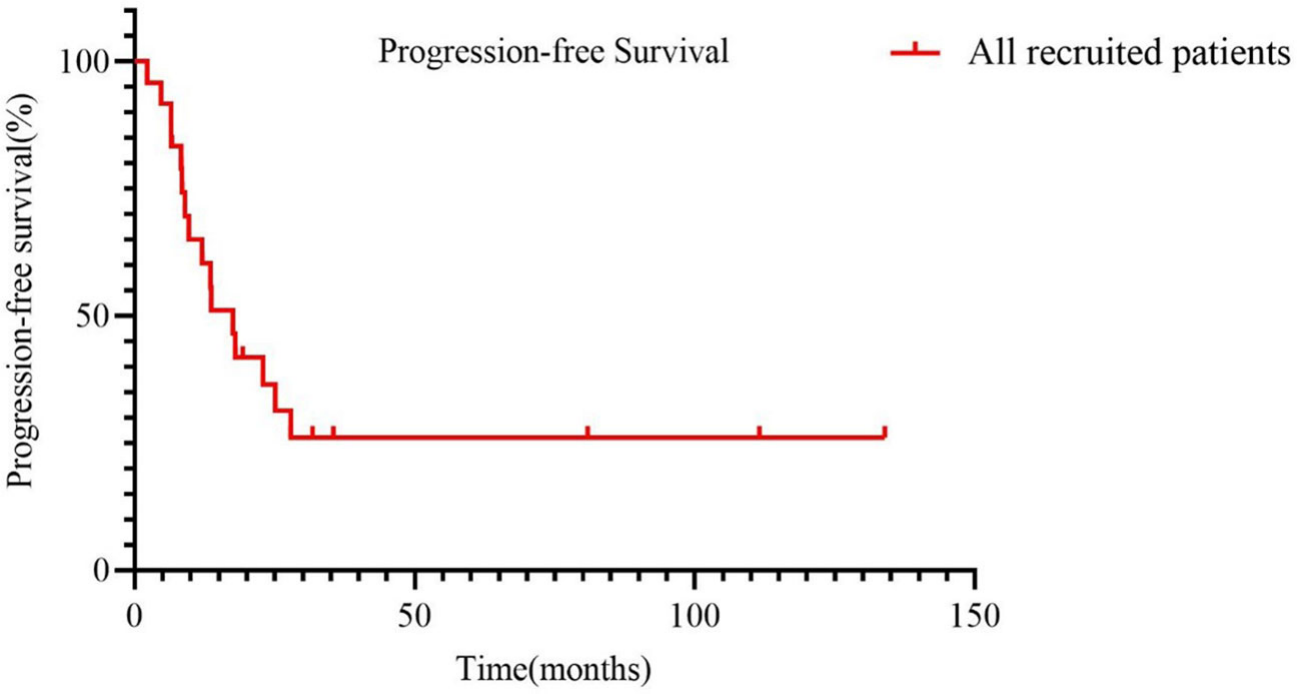
Progression-free survival of all enrolled patients.

**Figure 3:**
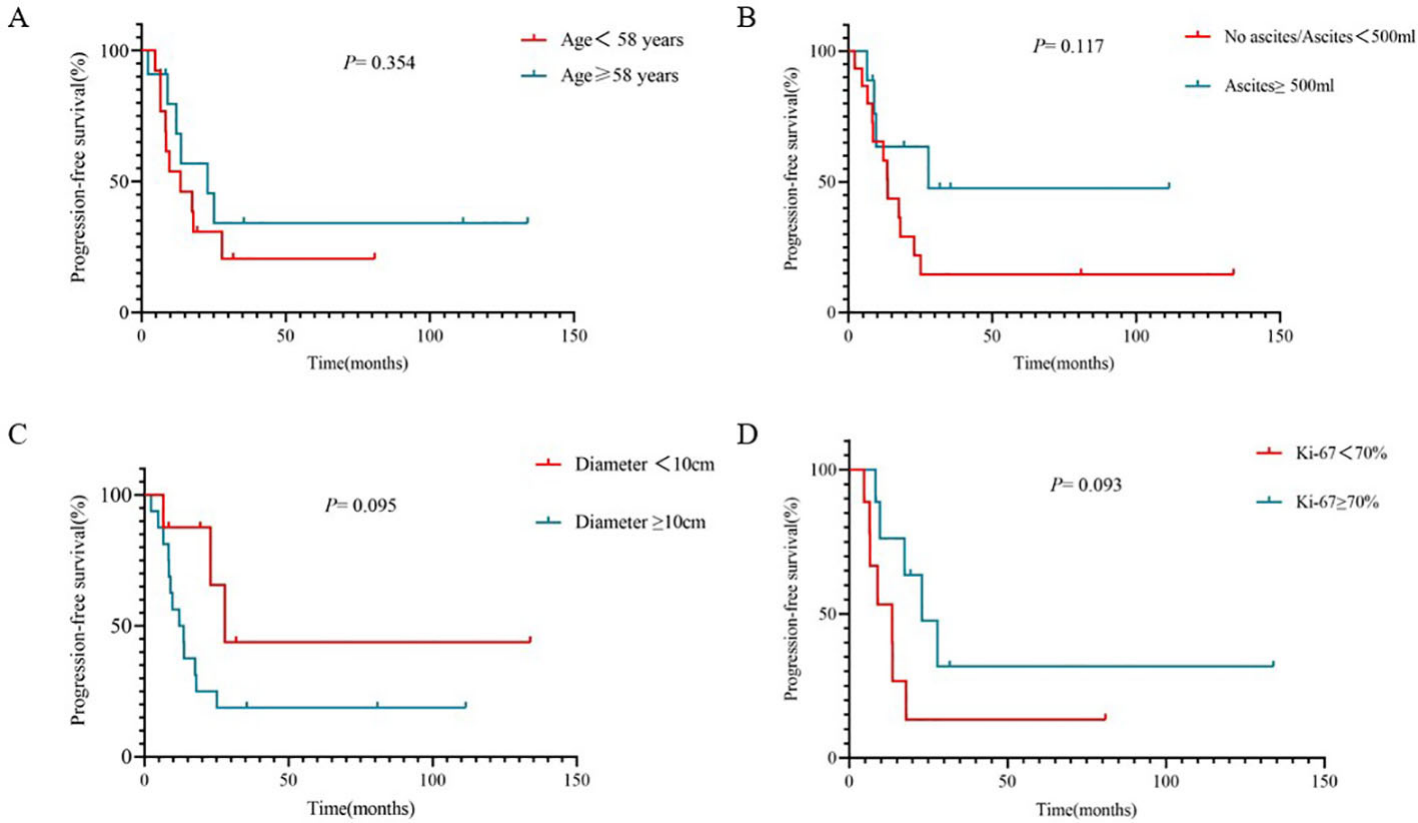
Kaplan-Meier curves were plotted for PFS with a log-rank Mantel-Cox test. PFS were compared by age at diagnosis **(A)**, volume of ascites **(B)**, maximum diameter of tumor **(C)** and Ki-67 index **(D)**.

For the entire cohort, the median progression-free survival was 17.53 months (**Figure 2**). Next, PFS were directly compared by age at diagnosis (one group was less than 58 years, and the other group was equal or order than 58 years). The median PFS of the two groups was 13.53 months and 22.93 months, respectively (HR 0.62, 95% CI 0.2339 -1.661, *p* =0.354, **Figure 3A**). In terms of the amount of ascites, patients were divided into no ascites/ascites <500ml and ascites ≥500ml. Kaplan-Meier curves were plotted for PFS for the two groups and comparison carried out with a log-rank Mantel-Cox test. The median PFS of the two groups was 13.7 months and 27.83 months, respectively (HR 0.72, 95% CI 0.1567-1.127, *p* =0.12, **Figure 3B**). Kaplan-Meier curves were similarly plotted for PFS for the maximum diameter of the tumor, and they were compared with a log-rank Mantel-Cox test. In comparing maximum diameter <10cm to maximum diameter ≥10 cm, the median PFS for maximum diameter <10cm was longer at 27.83 months compared to 12.80 months for maximum diameter ≥10 cm (HR 0.3626, 95% CI 0.1327-0.9906, *p* =0.095, **Figure 3C**). Given the potential that the Ki-67 index was related to the prognosis of OCS, we made one additional comparison: Ki-67 <70% compared to Ki-67 ≥70%. Interestingly, the median PFS of the two groups was 13.53 months and 22.93 months, respectively (HR 0.3938, 95% CI 0.1207 to 1.284, *p*= 0.093, **Figure 3D**).

Among all the included patients, 5 underwent genetic testing, 4 of whom were HRD-positive (2 of whom had BRCA mutation) and received first-line maintenance therapy with PARP inhibitors. One HRD-negative patient has not received PARPi maintenance therapy. The 4 patients who received maintenance therapy were all in the advanced stage (FIGO III-FIGO IV stage), 3 of whom received Olaparib and 1 received Niraparib. The median PFS of the 4 patients was 22.68 months. Among them, the patient with BRCA1 mutation had an HRD score of 96, which benefited the most and has not relapsed yet (**Table 2**).

**Table 2:** Summary of patients with PARP inhibitors as first-line maintenance therapy.

Patient number	FIGO stage	BRCA status	HRD status	Types of PARPi	PFS (months)	Current status
1	III	Negative	Positive	Olaparib	27.83	Recurrent
2	III	Negative	Positive	Niraparib	13.70	Recurrent
3	III	BRCA1 mutation	Positive	Olaparib	31.77	Not relapsed
4	IV	BRCA2 mutation	Positive	Olaparib	17.53	Recurrent

FIGO, International Federation of Gynecology and Obstetrics; HRD, homologous recombination deficiency; BRCA, BReast CAncer gene; PARPi Poly (ADP-ribose), polymerase inhibitor; PFS, Progression-free survival.

## 4 Discussion

This retrospective study reviewed patients diagnosed with OCS between March 2012 and October 2023 at our cancer center, reflecting the current status of ovarian carcinosarcoma treatment in China. Ovarian carcinosarcoma is extremely rare and there are few related studies including case reports, case series, or original articles with a small number of patients (4). Therefore, this study can provide a certain reference for clinical diagnosis and treatment. We have listed our findings and possible explanations in **Table 3**.

**Table 3:** Summary of potential prognostic factors for OCS in our study.

Prognostic factors	Better prognosis group	Potential biological mechanisms
Ascites	Ascites ≥500 ml	Epithelial components predominate.
Age	Age ≥58 years	More gene mutations.
Diameter of tumor	Diameter of tumor<10cm	Tumor burden influence prognosis.
Ki-67	Ki-67 ≥70%	High in tumor with epithelial components

Epithelial ovarian cancer (EOC) is the most common pathological type of ovarian cancer, while OCS represents a rare yet biologically unique ovarian cancer with a poorer prognosis (2). Therefore, we discuss and review the literature on the incidence and epidemiology, prognostic factors, and treatment strategies of OCS.

Our study showed an increase in the number of patients with OCS at our cancer center from 2012 to 2023. Similarly, A study from Korea also retrospectively analyzed the incidence, treatment, and prognosis of OCS in the Korean Central Cancer Registry from 1999 to 2018. Their nationwide registry-based study showed that the incidence of OCS also increased rapidly from 1999 to 2018 in Korea (4). However, the reason for the rapid increase in the incidence of OCS is still unclear. The rapid annual percent changes (APCs) of OCS may be due to the following hypotheses: First, because OCS shares similar histologic features, it can be considered another biphasic histology, such as mixed germ cell tumor. Recently, immunohistochemical markers, including SALL4 and CD10, have been developed as distinguishable markers for carcinosarcoma (22–25). Thus, with the advent of novel immunohistochemical markers, a number of OCS components have been identified and attributed to the growth of APCs. Second, a previous study from the National Cancer Institute’s SEER registry showed that demographic factors including increasing age, and unmarried status were more commonly associated with OCS (10).

There are some studies on which factors affect the prognosis of OCS. Gunjal Garg et al. revealed that age, stage, and lymphadenectomy were significant predictors in OCS (16). A study enrolled 37 patients reported that early FIGO stage was the only independent prognostic factor for survival, while histology (homologous/heterologous subtypes; grade, type or percentage of the epithelial component) had no significant impact on survival (18). Due to only 26 patients enrolled in our study, we have not found the relationship between FIGO stage and survival. This was similar with study reported by MA Harris et al. (19). Furthermore, order age at diagnosis has also been identified as a poor prognostic factor in patients with OCS (16). Different from the conclusion reported before, our real-world data showed that patients with age ≥58 years had a better prognosis than patients younger than 58 years, though the p value was not less than 0.05. This result needed to be explored and confirmed by further studies.

Some studies also revealed that the prognosis of OCS was linked to the residual disease and lymphadenectomy during the debulking surgery. Additionally, patients with bulk residual disease present after surgery was associated with a worse prognosis. Thus, optimal cytoreductive surgery plays a pivotal role in achieving a better prognosis (19, 20, 26).

In terms of lymphadenectomy, A 2010 study clearly support the beneficial effect of lymphadenectomy in OCS (HR 0.66, 95% CI 0.56-0.78) (16). Since most patients in our study achieved optimal cytoreductive surgery, the correlation between postoperative residual disease and prognosis have not been observed. In addition, few patients underwent lymphadenectomy, so the correlation between prognosis and lymph node dissection have not been found.

The histopathological characteristics and immunohistochemical molecular expressions may also be correlated with the prognosis of OCS. Several studies support the hypothesis that heterologous features (elements not normally present in the ovary) are associated with a worse prognosis (20, 26–28). R. ATHAVALE et al. revealed that stromal components adversely affected survival, and there was a trend to worse survival with serous compared with non-serous epithelial components (11). In addition, some studies have shown that vascular endothelial growth factor (VEGF) expression, p53 expression, Wilms tumor 1 (WT1) protein expression, and Ki-67 expression are correlated with the prognosis of patients (12–15, 29). In our study, we also compared prognosis of patients with different expression of Ki-67. Inconsistent with previous studies, our study found that patients with Ki67 <70% had a better prognostic trend than those with Ki67 ≥70%. This result may be caused by chemotherapy sensitivity in patients with different level of Ki67 expression. Interestingly, our real-world data also demonstrated that patients with no ascites/ascites <500ml had better prognosis than those with ascites ≥500ml. Furthermore, patients with diameter of tumor ≥10cm had a trend of worse outcomes than those with diameter <10cm. The reason for the above novel findings may be that the sarcoma component in ovarian cancer sarcoma accounts for a high proportion, resulting in no ascites or a small amount of ascites, which further affects the patient’s prognosis. As we all known, the FIGO stage of uterine sarcoma is related to the tumor diameter, which further verifies our similar finding in OCS. Certainly, in order to further confirm this, we need to conduct further research.

As for treatment strategies of OCS, optimal debulking surgery followed by chemotherapy has been frequently considered in the primary management of the disease, despite no available RCTs (30). A 2018 study showed that patients treated with carboplatin/paclitaxel had a longer median PFS than those treated with ifosfamide/paclitaxel for first-line chemotherapy (31). In our study, most of enrolled patients were also treated with platinum-based chemotherapy. Growing evidence has demonstrated the role of mutations of tumor biomarkers in diagnosing and treating epithelial ovarian cancer (32). Therefore, targeted therapies including bevacizumab and PARP inhibitors have been recommended for OCS, which are limited to case reports. Zhang et al. report a BRCAwt patient with advanced OCS who experienced a second and a third cytoreductive surgery in June 2017 and October 2019 and has been on niraparib maintenance therapy for more than 20 months after receiving second-line and third-line chemotherapy in 2021 (33). A 2023 study also showed that, genetic testing suggests that HRD-positive OCS with chemotherapy plus targeted therapy followed by treatment with a PARP inhibitor plus maintenance therapy may provide excellent efficacy and contribute to the patient’s long-term disease-free survival (34). In our study, we also had a case series of 4 patients treated with PARP inhibitors after genetic testing.

Nevertheless, our study also had some shortages. First, due to low incidence of OCS, we only enrolled 26 patients for 11 years at our cancer center. Second, some prognostic factors showed a predictive trend for PFS, but we have not acquired a significant p value due to small-scale cohort. Third, this was a retrospective study, which cannot be important as RCTs. Therefore, further research should be conducted in the future to provide stronger evidence for clinical diagnosis and treatment.

## 5 Conclusion

In conclusion, we conducted a real world study of diagnosis and treatment of OCS. Recently, the incidence of OCS is increasing with unknown reasons. Our study demonstrated that age at diagnosis, diameter of tumor, Ki-67 level, and volume of ascites may be the prognostic factors of OCS. Patients with HRD positive/BRCA mutation may benefit from PARP inhibitors.

## Data Availability

The raw data supporting the conclusions of this article will be made available by the authors, without undue reservation.
